# Beyond Foodborne HAV: Sexual Transmission Drives a New Wave of Cases in Romania

**DOI:** 10.3390/v18020215

**Published:** 2026-02-06

**Authors:** Adrian Paun, Irina Ianache, Ruxandra Moroti, Georgiana Pomohaci, Gratiela Tardei, Mike Youle, Simona Ruta, Cristiana Oprea

**Affiliations:** 1 Department of Infectious Diseases, “Carol Davila” University of Medicine and Pharmacy, 020021 Bucharest, Romania; adrian-marius.paun@rez.umfcd.ro (A.P.); georgiana.neagu@rez.umfcd.ro (G.P.); simona.ruta@umfcd.ro (S.R.); cristiana_oprea@umfcd.ro (C.O.); 2 School of Advanced Studies of the Romanian Academy (SCOSAAR), 010071 Bucharest, Romania; 3 “Victor Babeș” Clinical Hospital for Infectious and Tropical Diseases, 030303 Bucharest, Romania; gtardei@spitalulbabes.ro; 4 National Institute for Infectious Diseases “Matei Balș”, 021105 Bucharest, Romania; 5 Royal Free Hospital, London NW3 2QG, UK; mike@mikeyoule.com; 6 “Stefan S. Nicolau” Institute of Virology, 030304 Bucharest, Romania

**Keywords:** hepatitis A, HIV, MSM, coinfection, outbreak, sexually transmitted infections, sexual behaviors

## Abstract

**Background**: In 2022, Romania experienced a sharp increase in hepatitis A virus (HAV) infections, with evidence of predominant fecal–oral transmission through sexual contact, raising concern for an outbreak among men who have sex with men (MSM). **Methods**: We conducted a prospective multicenter study between 1 March 2022 and 1 March 2023 in two tertiary hospitals in Bucharest. HAV infection was defined by a compatible clinical presentation, elevated liver enzymes, and positive anti-HAV IgM serology. Clinical and laboratory characteristics were compared by transmission route and HIV status. **Results**: A total of 191 patients were diagnosed with HAV, including 105 MSM and 86 with foodborne transmission. All were unvaccinated. Most patients were male (82.2%), with a median age of 30 years (IQR 24–38). MSM were significantly younger and reported higher-risk sexual behaviors, including chemsex and multiple or occasional partners (*p* < 0.0001). Among MSM, 48 (25.1%) were living with HIV, most with preserved immune status and undetectable viral loads. Clinical manifestations were similar across groups, with jaundice being most frequent (89.5%). However, MSM exhibited more severe hepatocellular injury, reflected by higher ASAT and ALAT levels and lower prothrombin concentration, independent of HIV status. MSM were also more likely to have concomitant sexually transmitted infections, including syphilis and mpox (*p* < 0.001). Disease was predominantly mild, although MSM had longer hospital stays. **Conclusions**: The 2022 HAV surge in Romania was driven by both sexual and foodborne transmission. Targeted HAV vaccination, along with integrated sexual health services and harm-reduction strategies, is essential to prevent future outbreaks.

## 1. Introduction

Hepatitis A virus (HAV) is a major cause of acute viral hepatitis worldwide, with transmission occurring predominantly via the fecal–oral route or through close contact with infected individuals, including oral–anal sexual contact (rimming) [1,2]. Clinical manifestations range from asymptomatic infection to fulminant hepatitis, with children typically experiencing milder disease and adults presenting more severe forms [1]. Although it is a vaccine-preventable disease, more than 170 million new cases are diagnosed yearly [2].

According to the European Centre for Disease Prevention and Control (ECDC), in 2021, 30 European countries reported 3864 HAV cases (notification rate: 0.9/100,000) [3]. One year later, in 2022, the number of reported cases was even higher (4548, 1/100,000); the highest rates were reported in children aged 5–14 years [4]. However, over the last decade, several European countries have reported HAV outbreaks among men who have sex with men (MSM), often linked to low vaccination coverage. Recent clusters were reported in Hungary, Croatia, and Slovenia [2,5,6].

In Romania, HAV is a notifiable disease, and national surveillance data show a rate higher than the European average (4.5/100,000 in 2021), with most cases traditionally reported in children [3]. However, from early 2022, an increasing number of adult cases were observed, culminating in an outbreak that included both MSM (via fecal–oral transmission likely due to sexual contact) and the general population (via fecal–oral transmission due to contaminated food) [7].

The study aimed to describe the clinical and epidemiological features, laboratory findings, and outcomes in individuals diagnosed with HAV in two tertiary care centers in Bucharest during this outbreak and to compare subgroups according to possible transmission route, gender, and HIV status.

## 2. Materials and Methods

A prospective multicenter study was conducted between 1 March 2022 and 1 March 2023 at Victor Babes Hospital for Infectious and Tropical Diseases and Matei Bals National Institute for Infectious Diseases in Bucharest, Romania.

Inclusion criteria: Patients aged ≥18 years admitted with a confirmed diagnosis of acute hepatitis A were eligible.

HAV infection was suspected based on compatible clinical presentation (jaundice, fatigue, fever, nausea) together with elevated aminotransferases and was confirmed by positive anti-HAV IgM serology.

Disease severity: Clinical forms were classified according to prothrombin concentration (PC): mild (>70%), moderate (50–70%), severe (30–50%), and fulminant (<30%).

Data collection: Epidemiological data (age, gender, clustering of cases, probable transmission route, risk behaviors, vaccination status), HIV status, and clinical and biochemical parameters were extracted from electronic medical records. Information on past or concurrent sexually transmitted infections (STIs), sexual orientation, and sexual practices was obtained from patients’ medical histories. When relevant details were not documented in the electronic files, additional information was retrieved from the physicians involved in the patients’ care during hospitalization.

All patients with unknown status were tested for HIV and syphilis during hospitalization. Mpox testing was performed in case of suggestive symptoms. Screening for other STIs was not uniformly available to all patients.

For all patients, the underlying mechanism was considered fecal–oral. For analytical purposes, the cohort was divided into two mutually exclusive exposure groups:

1. The MSM group, comprising individuals reporting sexual exposure with male partners;
2. The non-MSM group, comprising individuals who explicitly denied MSM behavior or any sexual risk exposure. Non-MSM patients were a priori classified as having foodborne transmission (FBT), based on the absence of sexual risk behavior and the presence of intrafamilial or small-group epidemiological links when identifiable. These small-group clusters involved a heterogeneous age and gender composition and did not correspond to MSM sexual networks. Other non-MSM individuals reported purchasing food from supermarkets, including the salads suspected during the contemporaneous foodborne outbreak, or food originating from regions where similar HAV cases were reported, including areas near the capital with documented water-supply problems. Although a direct food-exposure source could not be established for individual patients, all non-MSM individuals had epidemiological contexts consistent with non-sexual fecal–oral transmission and were therefore included in the FBT group.

Molecular genotyping was unavailable.

Written informed consent for data collection was signed by all patients.

Statistical analysis: Analyses were performed using IBM SPSS Statistics 26.0 and Microsoft Excel 365. Normality was assessed using the Shapiro–Wilk and Kolmogorov–Smirnov tests. Continuous variables were expressed as means ± SD or medians (IQR), and categorical variables as percentages. Group comparisons employed Student’s t-test or ANOVA for normally distributed variables, Mann–Whitney U or Kruskal–Wallis for nonparametric distributions, and χ^2^ or Fisher’s exact test for categorical data. Correlations were assessed with Pearson’s or Spearman’s coefficients. A *p*-value < 0.05 was considered statistically significant.

## 3. Results

### 3.1. HAV Outbreak

#### 3.1.1. Demographics and Epidemiology

Between March 2022 and March 2023, 191 individuals with laboratory-confirmed acute hepatitis A were hospitalized in the two tertiary care centers included in the study.

All 191 individuals with HAV were unvaccinated.

Of 191 patients, 82.2% were male, with a median age of 30 years [IQR 24–38].

Based on sexual orientation and practices, 105 were confirmed MSM, and the other 86 entered the FBT group.

All MSM were from urban areas, whereas 93% of the FBT group lived in urban areas.

Patients in the MSM group were significantly younger than those in the FBT group (median age 30 [IQR 24–36] vs. 35 [IQR 26–50], *p* = 0.002).

Reported risk behaviors among MSM included chemsex practices (26.5%, 26/98) and multiple or occasional sexual partners (56.1%, 55/98) (*p* < 0.0001). Among the 26 patients disclosing chemsex, intranasal or intravenous mephedrone and oral gamma-hydroxybutyric acid were the most frequently used substances.

Forty-eight patients (25.1%) were MSM living with HIV (*p* < 0.0001), of whom 42 (93.3%) had undetectable viral loads while receiving antiretroviral therapy (Table 1). The median CD4+ T-cell count in this subgroup was 609 cells/μL (IQR 464–865). Additionally, six individuals received a new HIV diagnosis during the HAV outbreak.

#### 3.1.2. Clinical and Laboratory Findings

Jaundice was the most common clinical manifestation, occurring in almost all patients (89.5%), followed by nausea (68.5%), fatigue (55.5%), and fever (50.8%). No statistically significant differences in clinical presentations were observed according to the mode of HAV transmission or HIV status (Table 2).

MSM exhibited more severe hepatocellular injury, with significantly higher ASAT (*p* = 0.01) and ALAT (*p* = 0.05) levels, while no significant differences were observed in cholestatic parameters, including bilirubin. MSM also had a significantly lower prothrombin concentration (*p* = 0.045). Aside from these findings, no other major clinical or laboratory differences were identified between PLWH and those without HIV (Table 3).

#### 3.1.3. Coinfections with STIs

Sexually transmissible coinfections, including syphilis (n = 32), mpox (n = 10), and other STIs (n = 19), were significantly more frequent among MSM (*p* < 0.001).

#### 3.1.4. Outcomes

Based on clinical outcomes and biochemical markers, 97 cases (50.7%) were classified as mild, 58 (30.3%) as moderate, 25 (13.1%) as severe, and 11 (5.7%) as fulminant.

The median hospitalization duration was 10 days [IQR 7–12]. The MSM group had a significantly longer hospital stay (9 days [IQR 7–12] vs. 8 days [IQR 6–11], *p* = 0.036). Overall outcomes were favorable for all patients except one, who developed sepsis and subsequently died.

## 4. Discussion

This study describes one of the first documented hepatitis A virus (HAV) outbreaks in Romania involving dual transmission pathways: sexual transmission among MSM and foodborne transmission within the general population.

In our cohort, MSM patients exhibited significantly greater hepatocellular injury compared with non-MSM individuals. This finding does not appear to be explained by HIV-related immunosuppression, as people living with HIV in our study were on antiretroviral therapy, had undetectable viral loads, and demonstrated good immunological status, with a median CD4 count above 500 cells/μL. Moreover, not all MSM in the cohort were living with HIV, indicating that the observed differences in liver injury were not solely attributable to HIV infection. Other mechanisms may therefore be involved. One plausible explanation is a higher infectious inoculum associated with specific sexual practices that facilitate fecal–oral HAV transmission, a pattern also described in other HAV outbreaks among MSM.

Although concurrent STIs could theoretically contribute to increased hepatic inflammation [7], STI screening was not uniformly available, limiting our ability to perform a detailed subgroup analysis. This limitation has been acknowledged, and future studies incorporating systematic STI testing and molecular epidemiology may help clarify the factors underlying the more pronounced hepatocellular injury observed in MSM.

Our findings provide one of the first documented insights into chemsex-related transmission dynamics in Romania. We identified a cluster of MSM, most living with HIV, who were epidemiologically linked through recurrent group sex and chemsex events and who acquired HAV during the study interval. Among those disclosing chemsex practices, intranasal or intravenous mephedrone and oral gamma-hydroxybutyric acid were most frequently used substances. Several individuals from this network were simultaneously diagnosed with mpox, illustrating how sexualized drug use can facilitate rapid dissemination of multiple sexually associated infections. These observations highlight the need for targeted harm-reduction strategies, chemsex-specific counseling, and enhanced HAV vaccination coverage among MSM, especially among PLWH [8,9,10,11]. Most individuals from both transmission pathways reside in urban areas, suggesting increased exposure related to population density, social mixing patterns, and behavioral trends typical of metropolitan settings. During the study period, national media and public health authorities reported detection of HAV in ready-to-eat vegetable products distributed by large supermarket chains. Although a direct epidemiologic link to our cohort cannot be established, these events raise concerns that contaminated fresh produce items may have contributed to the rise in foodborne cases. The identification of individuals with HAV infection living in rural areas—exclusively associated with unsafe water or food sources—reinforces the need for improved public health control measures and the active engagement of local authorities. The epidemiological data available to us did not allow detailed reconstruction of cluster linkages for either the MSM or the FBT transmission groups. As specified in the Section 2, molecular genotyping was unavailable during the study period. This important limitation affects the interpretation of our findings, as the absence of sequencing data prevented confirmation of transmission links or identification of specific outbreak strains.

In line with observations from other European countries, MSM in our study demonstrated high rates of HIV and syphilis coinfection, reflecting overlapping transmission pathways and high-risk sexual behaviors such as chemsex, condomless intercourse, and multiple partnerships [7,12,13]. Reports from different parts of the world describe concurrent hepatitis A and mpox post-pandemic outbreaks among MSM, suggesting possible clusters of transmission. Most data come from Western Europe, but in recent years, Central and Eastern European countries have reported similar outbreaks. Instances of concomitant or sequential HIV, mpox, other STIs, and HAV diagnoses support the notion that these infections share risk factors, acquisition modes, and potential for heightened clinical severity [7,12,13]. Routine screening for HIV and STIs in all patients with HAV or mpox should be considered essential to enable early therapeutic engagement and reduce complications. Although sexually linked HAV outbreaks have been documented in several European countries [2,5,6,10,11,14,15,16,17], such as Croatia, Hungary, France, Spain, and Germany, Romanian data regarding HAV and mpox outbreaks among MSM remain scarce [7,13].

In early 2022, one of our centers observed a marked increase in HAV among MSM, culminating in a report of 35 cases in August 2022, signaling the onset of a national outbreak [13]. Other Romanian reports have since confirmed continued increases in HAV incidence among MSM [7]. Our centers also documented overlapping HAV and mpox outbreaks in the capital city, Bucharest [12,13], with 42 mpox diagnoses during the study period, seven of them simultaneously with HAV. Chemsex use, reported by one quarter of MSM in our cohort, is consistent with emerging but limited data describing this behavior among Romanian MSM [12,13]. As chemsex practices become more widespread, healthcare systems must strengthen their capacity to address associated infectious and non-infectious risks. The recent inclusion of chemsex-related recommendations in the European AIDS Clinical Society (EACS) guidelines reflects growing recognition of its clinical importance [18]. Although our sample size limits definitive conclusions, the more severe clinical outcomes observed in individuals coinfected with HAV and mpox raise the possibility of synergistic pathogenic effects and should prompt further investigation. The absence of significant differences in HAV severity between PLWH and PLWoH is consistent with findings from other cohorts, particularly among PLWH receiving suppressive antiretroviral therapy with preserved CD4 counts. Nevertheless, the clustering of newly diagnosed HIV infections among individuals presenting with HAV suggests that HAV may serve as a sentinel marker for identifying persons at increased risk of HIV acquisition [12]. This outbreak underscores substantial gaps in HAV vaccination coverage in Romania. Despite the availability of effective vaccines, HAV immunization is not routinely offered to high-risk populations such as MSM and PLWH. Limited sexual health education and insufficient harm-reduction programs further increase vulnerability to recurrent outbreaks. Strengthening vaccination delivery, particularly in HIV clinics and through primary care providers, alongside comprehensive sexual health education for young people and MSM, is critical for preventing future HAV epidemics [11].

## 5. Conclusions

To our knowledge, this is one of the first reports about an HAV outbreak in Romania affecting MSM, which describes in detail the sexually transmitted HAV. MSM exhibited significantly greater hepatocellular injury, a difference that appears to be related primarily to MSM-specific transmission dynamics rather than to HIV status. This pattern may reflect exposure to a higher viral inoculum associated with oral–anal sexual practices, which has been described in other MSM-associated HAV outbreaks. Although concomitant STIs, including HIV, may contribute to hepatic inflammation in some individuals, the increased severity observed in our cohort was present even among MSM without HIV infection, supporting the hypothesis that inoculum size plays a central role.

Strengthening HAV vaccination programs—particularly in HIV and STIs clinics and through primary care—is urgently required. Comprehensive sexual education and targeted harm-reduction initiatives for young people and MSM are essential for preventing future outbreaks.

## Figures and Tables

**Table 1:** Demographics and risk factors in patients with HAV.

Characteristics	Total n = 191	MSM n = 105	FBT n = 86	*p*-Value
**Male gender at birth n (%)**	158 (82.7)	105 (100.0)	53 (61.6)	0.083
**Median age (years) [IQR]**	30 [24–38]	30 [24–36]	35 [26–50]	**0.002**
**Nr. of PLWH n (%)**	48 (25.1)	48 (48.9)	0 (0.0)	**0.0001**
**Nr of persons with risk behaviors**				
**Chemsex n (%)**	26 (13.6)	26 (24.7)	0 (0.0)	**0.0001**
**Multiple/occasional partners n (%)**	55 (28.7)	55 (52.3)	0 (0.0)	**0.0001**

Legend: PLWH = people living with HIV; MSM = men who have sex with men; FBT = foodborne transmission.

**Table 2:** Symptoms of HAV patients—comparison based on modes of transmission and on HIV status.

Symptoms	Total n = 191	MSM n = 105	FBT n = 86	*p*-Value MSM vs. FBT	PLWH n = 48	PLwoH n = 143	*p*-Value PLWH vs. PLwoH
**Jaundice** n (%)	171 (89.5)	95 (90.4)	76 (88.3)	0.525	48 (100.0)	123 (86.0)	0.960
**Nausea** n (%)	130 (68.0)	72 (68.5)	58 (67.4)	0.128	36 (75.0)	94 (65.7)	0.783
**Fatigue** n (%)	106 (55.4)	64 (60.9)	42 (48.8)	0.365	33 (68.75)	73 (51.0)	0.115
**Fever** n (%)	96 (50.2)	53 (50.4)	43 (50.0)	0.525	27 (56.25)	69 (48.2)	0.981
**Abdominal pain** n (%)	76 (39.7)	47 (44.7)	29 (33.7)	0.307	26 (54.16)	50 (34.9)	0.202
**Vomiting** n (%)	78 (40.8)	45 (42.8)	33 (38.3)	0.034	19 (39.5)	59 (41.2)	0.611
**Loss of appetite** n (%)	76 (39.7)	41 (39.0)	35 (40.6)	0.237	17 (35.4)	59 (41.2)	0.141
**Headache** n (%)	32 (16.7)	20 (19.0)	12 (13.9)	0.237	10 (20.8)	22 (15.3)	0.771
**Myalgia** n (%)	34 (17.8)	17 (16.1)	17 (19.7)	0.349	7 (14.5)	27 (18.8)	0.636

Legend: MSM = men who have sex with men; FBT = foodborne transmission; PLWH = people living with HIV; PLwoH = people living without HIV.

**Table 3:** Laboratory findings at admission—comparison based on modes of HAV acquisition.

Variable	Total n = 191	MSM n = 105	FBT n = 86	*p*-Value
**ALAT (IU/L)** **(range)**	2840 (1652–4448)	3333 (2111–4977)	2115 (1181–2824)	**0.002**
**ASAT (IU/L)** **(range)**	1477 (527–2723)	1650 (878–2994)	1058 (242–2272)	**0.032**
**Total bilirubin (mg/dL)** **(range)**	8.38 (5.7–11.5)	9.4 (6.6–12.5)	7.37 (5.2–10.1)	0.927
**Direct bilirubin (mg/dL)** **(range)**	7.5 (5.0–10.2)	8.6 (5.6–10.7)	6.55 (4.3–9.3)	0.350
**Prothrombin (%)** **(range)**	72 (57.0–86.0)	67 (52.0–80.5)	75.5 (64.7–88.7)	**0.045**

Legend: All values are expressed as the median, with their range in brackets. N = normal values (reference interval lab values). ALAT = alanine aminotransferase (N: 6–63 IU/L); ASAT = aspartate aminotransferase (N: 0–38 IU/L); total bilirubin (N: 0–1.1 mg/dL); direct bilirubin (N: 0–0.3 md/dL); prothrombin (%) = prothrombin concentration (N: 80–100%).

## Data Availability

The data presented in this article are available upon request from the corresponding authors. The raw patient data are preserved in the electronic database of Victor Babes Clinical Hospital for Infectious and Tropical Diseases and at the National Institute for Infectious Diseases Matei Bals, Bucharest, Romania, and consist of their files (diagnosis, history, outcome, and all laboratory tests).

## Abbreviations

The following abbreviations are used in this manuscript:

ALAT	Alanine aminotransferase
ASAT	Aspart aminotransferase
HAV	Hepatitis A virus/Hepatitis A
HIV	Human immunodeficiency virus
ECDC	European Centre for Disease Prevention and Control
EACS	European AIDS Clinical Society
FBT	Foodborne transmission
MSM	Men who have sex with men
PLWH	People living with HIV
PLwoH	People living without HIV
